# The Effect of *XPD* Polymorphisms on Digestive Tract Cancers Risk: A Meta-Analysis

**DOI:** 10.1371/journal.pone.0096301

**Published:** 2014-05-02

**Authors:** Haina Du, Nannan Guo, Bin Shi, Qian Zhang, Zhipeng Chen, Kai Lu, Yongqian Shu, Tao Chen, Lingjun Zhu

**Affiliations:** 1 Department of Oncology, The First Affiliated Hospital of Nanjing Medical University, Nanjing, China; 2 Department of Oncology, The first people's Hospital of Zhangjiagang City, Suzhou, China; 3 Department of Gastrointestinal Surgery, The First Affiliated Hospital of Nanjing Medical University, Nanjing, China; University of Pittsburgh, United States of America

## Abstract

**Background:**

The Xeroderma pigmento-sum group D gene (*XPD*) plays a key role in nucleotide excision repair. Single nucleotide polymorphisms (SNP) located in its functional region may alter DNA repair capacity phenotype and cancer risk. Many studies have demonstrated that *XPD* polymorphisms are significantly associated with digestive tract cancers risk, but the results are inconsistent. We conducted a comprehensive meta-analysis to assess the association between *XPD* Lys751Gln polymorphism and digestive tract cancers risk. The digestive tract cancers that our study referred to, includes oral cancer, esophageal cancer, gastric cancer and colorectal cancer.

**Methods:**

We searched PubMed and EmBase up to December 31, 2012 to identify eligible studies. A total of 37 case-control studies including 9027 cases and 16072 controls were involved in this meta-analysis. Statistical analyses were performed with Stata software (version 11.0, USA). Odds ratios (ORs) with 95% confidence intervals (CIs) were used to assess the strength of the association.

**Results:**

The results showed that *XPD* Lys751Gln polymorphism was associated with the increased risk of digestive tract cancers (homozygote comparison (GlnGln *vs.* LysLys): OR = 1.12, 95% CI = 1.01–1.24, *P* = 0.029, *P*_heterogeneity_ = 0.133). We found no statistical evidence for a significantly increased digestive tract cancers risk in the other genetic models. In the subgroup analysis, we also found the homozygote comparison increased the susceptibility of Asian population (OR = 1.28, 95% CI = 1.01–1.63, *P* = 0.045, *P*_heterogeneity_ = 0.287). Stratified by cancer type and source of control, no significantly increased cancer risk was found in these subgroups. Additionally, risk estimates from hospital-based studies and esophageal studies were heterogeneous.

**Conclusions:**

Our meta-analysis suggested that the *XPD* 751Gln/Gln genotype was a low-penetrate risk factor for developing digestive tract cancers, especially in Asian populations.

## Introduction

Digestive tract cancers, especially gastric, esophageal and colorectal cancers, are a major global health problem. Globocan data in 2008 showed [1] that the standardized incidence of colorectal cancer, gastric cancer and esophageal cancer were located in 4th, 6th and 9th in all tumors, respectively. The standardized mortality rate of gastric cancer, coming after lung cancer and breast cancer, ranked the third place. Moreover, colorectal cancer and esophageal cancer also ranked top ten in cancer mortality rankings. The incidence of different cancer varies widely among different racial and ethnic groups which may be partly attributed to lifestyle and genetic background [2]. Exposure to environmental carcinogens can cause different types of DNA damage that subsequently lead to carcinogenesis of different tissues, if left unrepaired [3].

DNA repair mechanisms, such as the nucleotide excision repair (NER), base excision repair pathway (BER) and double-strand break pathway, are essential for maintaining genome integrity and preventing carcinogenesis. NER, the most versatile, well studied DNA repair mechanism in humans, is mainly responsible for repairing bulky DNA damage, such as DNA adducts caused by UV radiation, mutagenic chemicals, or chemotherapeutic drugs [4]. The major component of NER, xeroderma pigmentosum group D (*XPD* or *ERCC2*), mapped in chromosome 19q13.3, spans over 20 kb, contains 23exons and encodes the 761-amino acid protein. It has two functions: nucleotide excision repair and basal transcription as part of the transcription factor complex (TFIIH) [5]. Mutations on different sites in *XPD* gene can give rise to repair and transcription defects, and altered DNA repair capacity can render a higher risk of developing different types of cancer [5]–[11]. Several polymorphisms of *XPD* were identified, like Asp312Asn, Lys751Gln, Arg194Trp and Arg399Gln. The XPD polymorphic loci that has been of particular interest in molecular epidemiology studies is the Lys751Gln polymorphism (rs13181) in exon 23 [12]. The lysine to glutamine transition at position 751 in exon 23 may affect different protein interactions, diminish the activity of TFIIH complexes, and alter the genetic susceptibility to cancer [13].

Genetic variant in *XPD* Lys751Gln had been demonstrated to be associated with some cancers risk in different meta-analysis, such as esophageal cancer, gastric cancer, colorectal cancer, breast cancer, prostate cancer, lung cancer and bladder cancer [14]–[23]. However, due to an insufficient number of publications, they did not calculate pooled odds ratios (ORs) of digestive tract cancers comprehensively. In consideration of the extensive role of *XPD* in digestive tract cancers, we performed a meta-analysis of all 37 eligible case–control studies: oral cancer, esophageal cancer, gastric cancer http://www.sciencedirect.com/science/article/pii/S0188440911000853 - bib10and colorectal cancer, to derive a more precise association of *XPD* Lys751Gln polymorphism and different types of digestive tract cancers risk.

## Materials and Methods

### Identification of eligible studies

Using PubMed, we identified all published case–control studies which investigated the association between the *XPD* Lys751Gln polymorphism and digestive tract cancers risk using a retrieving query formulation “(*XPD* or ERCC2) polymorphisms AND (colorectal cancer OR gastric cancer OR esophageal cancer OR oral cancer)”.The digestive tract cancers in this article refer to oral cancer, esophageal cancer, gastric cancer and colorectal cancer. We also searched references in published articles and reviews on this topic in PubMed. Eligible studies had to meet the following criteria: (a) only case-control designs were considered, (b) The study explored the correlation between different types of digestive tract cancers and *XPD* Lys751Gln polymorphism. Major exclusion criteria were (a) no control population, (b) no available genotype frequency. (c) Genotypic distribution of the controls was not in agreement with Hardy-Weinberg equilibrium (HWE). (d) Duplication of the previous publications, the largest or most recent publication was selected.

### Data Extraction

Information was carefully extracted from all eligible publications independently by two authors according to the inclusion criteria listed above. If the two pieces of typed data were different, a third investigator would be asked to check and to make sure all data were right. The following information was extracted from each study: first author, year of publication, country of study population, ethnicity, source of controls, number of cases and controls with different genotypes and HWE (Table 1).

**Table 1:** Characteristics of XPD polymorphisms Included in the Meta-analysis.

study	Year	Ethnicity	Source of controls	Cases				Controls				*P* for HWE
				N	Genotypes			N	Genotypes			
					Lys/Lys	Lys/Gln	Gln/Gln		Lys/Lys	Lys/Gln	Gln/Gln	
**Oral cancer**												
Surya	2005	Asian	PB	110	49	46	15	110	71	31	8	0.09
Da-Tian	2007	Asian	HB	154	134	18	2	105	89	15	1	0.68
Mousumi	2007	Asian	HB	388	190	158	40	309	158	125	26	0.85
Suparp	2005	Asian	PB	105	83	21	1	164	126	36	2	0.74
**Esophageal cancer**												
Xing	2002	Asian	HB	433	367	63	3	524	451	70	3	0.87
Xing	2003	Asian	HB	325	278	44	3	383	331	49	3	0.43
Yu	2004	Asian	HB	135	108	16	11	152	133	17	2	0.10
Alan	2005	European	HB	56	31	21	4	95	34	46	15	0.93
Ye	2006	European	PB	303	99	156	48	472	198	203	71	0.11
Geoffrey	2007	European	HB	182	61	98	23	336	143	161	32	0.16
Ranbir	2007	Asian	HB	120	52	61	7	160	63	77	20	0.63
Darren	2008	European	HB	312	104	159	49	453	193	208	52	0.72
Heather	2008	European	PB	208	80	94	34	247	91	121	35	0.60
James	2008	European	PB	263	108	123	32	1337	575	588	174	0.22
Jennifer	2009	European	HB	346	137	153	56	456	187	216	53	0.43
Zhai	2009	Asian	HB	200	167	31	2	200	148	51	1	0.12
Huang	2012	Asian	HB	213	150	55	8	358	274	79	5	0.79
**Gastric cancer**												
Huang^a^	2005	European	PB	279	381	107	126	46	145	163	73	0.03
Lou	2006	Asians	HB	238	205	30	3	200	164	33	3	0.38
Ye	2006	European	PB	126	49	61	16	472	198	203	71	0.11
Ruzzo	2007	European	PB	89	29	44	16	94	25	53	16	0.18
Zhou	2006	Asians	PB	253	224	26	3	612	522	86	4	0.82
Gabriel	2008	European	HB	245	99	105	41	1172	447	555	170	0.91
Doecke	2008	European	PB	303	127	140	36	1337	575	588	174	0.22
Zhang	2009	Asians	PB	207	166	39	2	212	172	39	1	0.43
Domenico	2010	European	PB	295	90	157	48	546	177	284	85	0.09
EMEL	2010	European	PB	40	14	18	8	247	102	114	31	0.92
Long^a^	2010	Asians	HB	361	616	139	151	71	400	164	52	0.00
Ayse^a^	2011	European	HB	106	116	30	56	20	40	43	33	0.01
**Colorectal cancer**												
Camilla	2006	Asians	PB	105	43	47	15	331	148	142	41	0.44
Mariana	2006	European	PB	740	387	298	55	789	392	317	80	0.18
Skjelbred	2006	European	PB	156	58	76	22	398	175	173	50	0.48
Victor	2006	European	HB	357	158	150	49	318	135	145	38	0.92
Mariana	2007	Asians	PB	303	251	48	4	1163	998	159	6	0.90
Chih-Ching	2007	Asians	HB	717	602	112	3	731	631	96	4	0.86
Rikke	2007	European	PB	396	160	178	58	798	311	382	105	0.47
Tomasz	2009	European	HB	100	56	33	11	100	42	41	17	0.21
Wang	2010	Asians	HB	302	138	130	34	291	137	117	37	0.13
Jelonek	2010	European	PB	123	54	47	22	153	66	68	19	0.81
Canbay	2011	European	PB	79	31	37	11	247	102	114	31	0.92

HB: hospital based.

PB: population based.

a : Hardy–Weinberg Equilibrium (HWE) in controls: *P*<0.05. Overall analysis and subgroup analysis does not include these studies' data.

### Statistical Analysis

We assessed the departure from the Hardy–Weinberg equilibrium for the control group in each study using Pearson's goodness-of-fit χ^2^test with 1 degree of freedom. Heterogeneity among studies was checked by the random-effects model (the Der Simonian and Laird method) if there was significant heterogeneity [24]. A *P* value of more than the nominal level of 0.05 for the Q statistic indicated a lack of heterogeneity across studies, allowing for the use of the fixed -effects model (the Mantel–Haenszel method) [25]. If *P* value less than 0.05 was considered as having heterogeneity, the results can not be pooled together and discussed. The risks ORs of digestive tract cancers associated with the *XPD* Lys751Gln polymorphism were estimated for each study. The pooled ORs were evaluated on co-dominant model (Lys/Gln *vs*.Lys/Lys, Gln/Gln *vs*. Lys/Lys), dominant model (Gln/Gln + Lys/Gln *vs*. Lys/Lys), recessive model (Gln/Gln *vs*. Lys/Gln+Lys/Lys), respectively. Subgroup analyses were performed by cancer types, ethnicity and source of controls. The publication bias was diagnosed by the funnel plot, in which the standard error of log (OR) of each study was plotted against its log (OR). Funnel plot asymmetry was assessed by Egger's linear regression test. The significance of the intercept was determined by the t test suggested by Egger (*P*<0.05 was considered representative of statistically significant publication bias) [26]. All the statistical tests were performed with STATA version11.0 (Stata Corporation, College Station, TX, USA).

## Results

### Study characteristic

**Figure 1:**
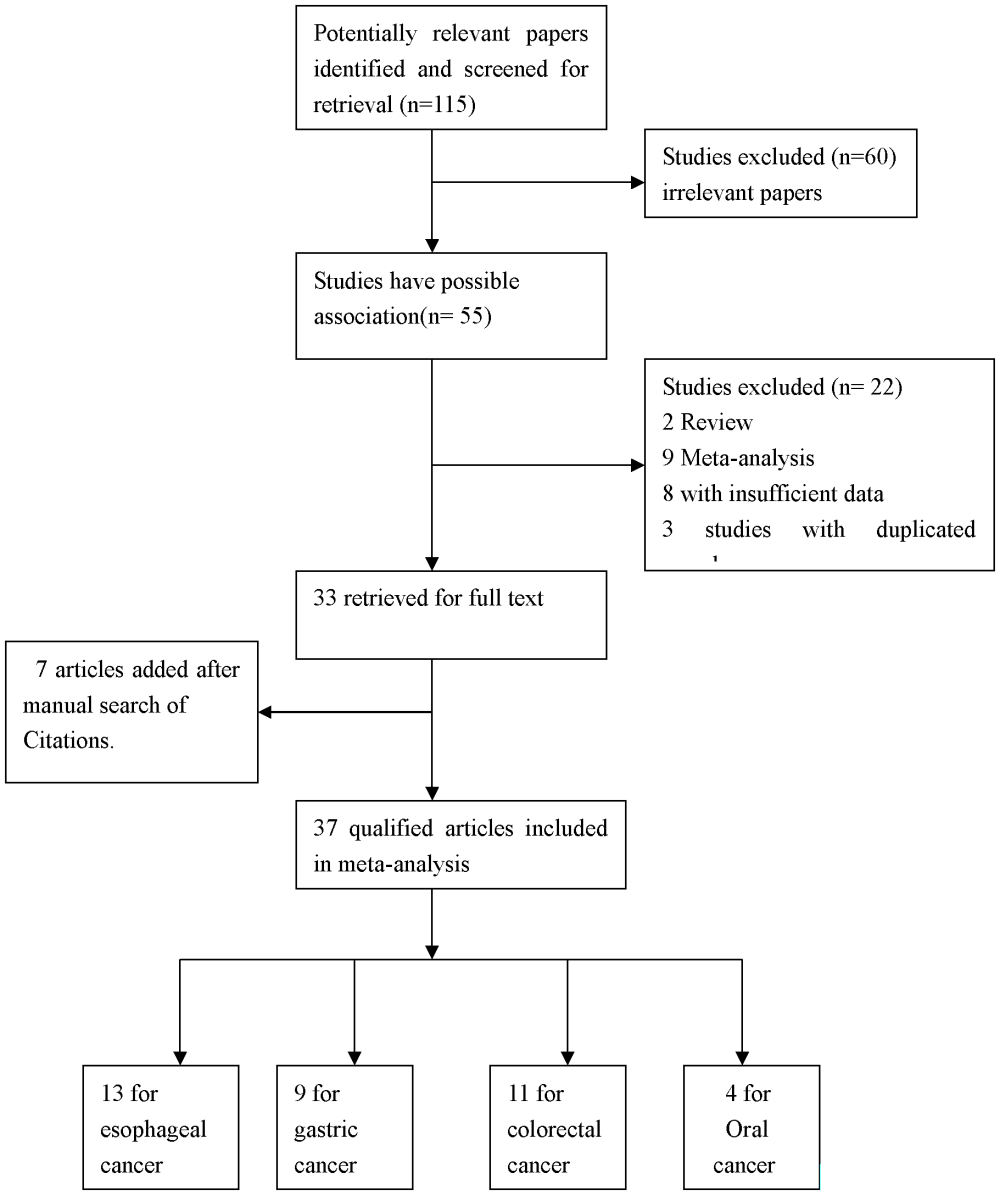
Flow diagram of studies identification.

A total of 107 potential relevant studies were retrieved through PubMed (Figure 1). After carefully reviewing, 40 eligible case-control studies (3 studies not consistent with HWE were also shown) on the relationship between *XPD* Lys715Gln polymorphism and digestive cancers risk were involved in this meta-analysis, including 4 oral cancer studies [62]–[65], 13 esophageal cancer studies [27]–[39], 12 gastric cancer studies [36], [40]–[50] and 11 colorectal cancer studies [51]–[61]. As shown in Table 1, 17 studies were conducted in Asians, 20 studies in Europeans. In addition, there were 18 hospital-based studies, 19 population-based studies. Diverse genotyping methods were used, including PCR-RFLP, PCR-SSCP, Taqman, Real-time PCR and SEB PCR. All studies indicated that the genotypic distribution of the controls were consistent with HWE.

### Meta-analysis

**Figure 2:**
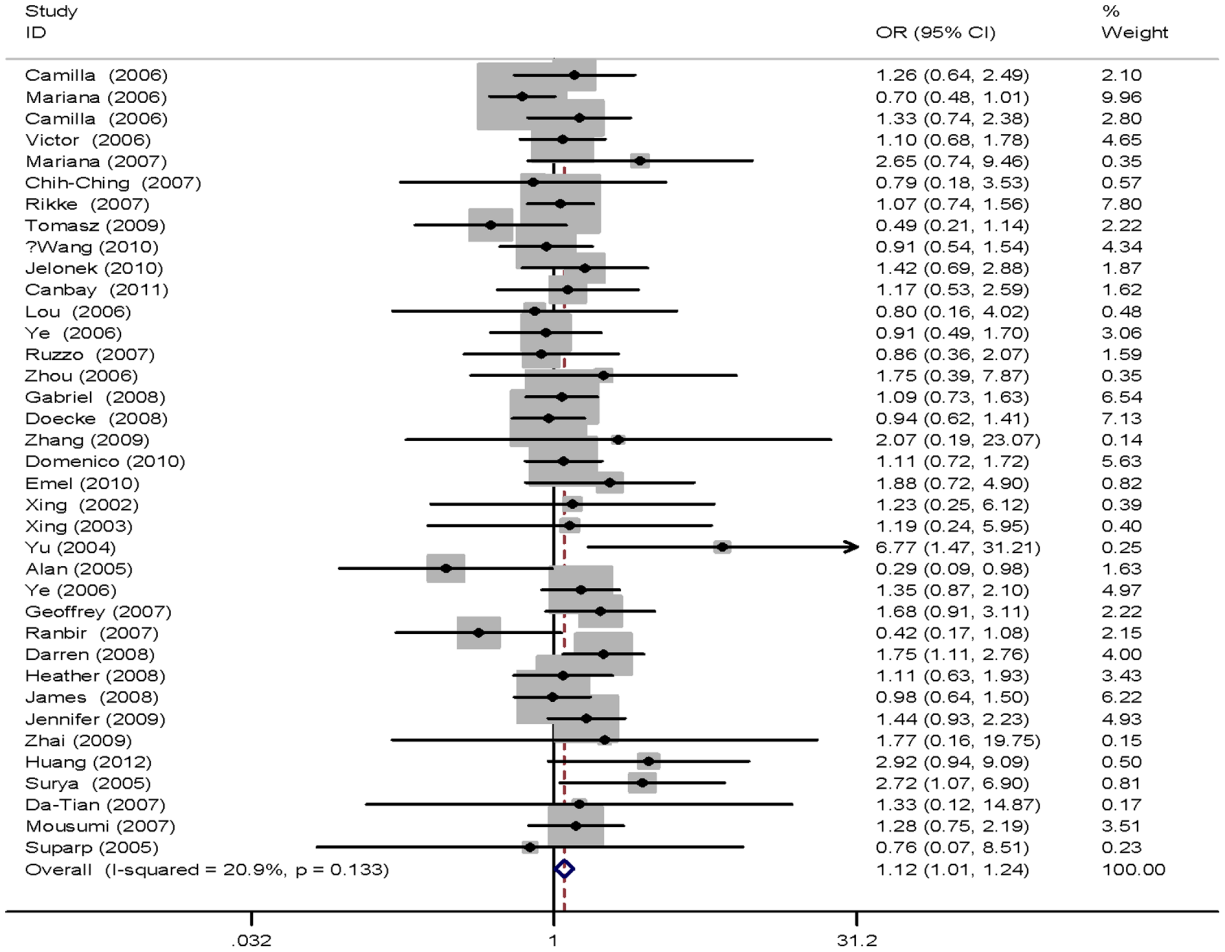
Forest plot of digestive cancer risk associated with the XPD Lys751Gln polymorphisms. Homozygote comparison.

**Figure 3:**
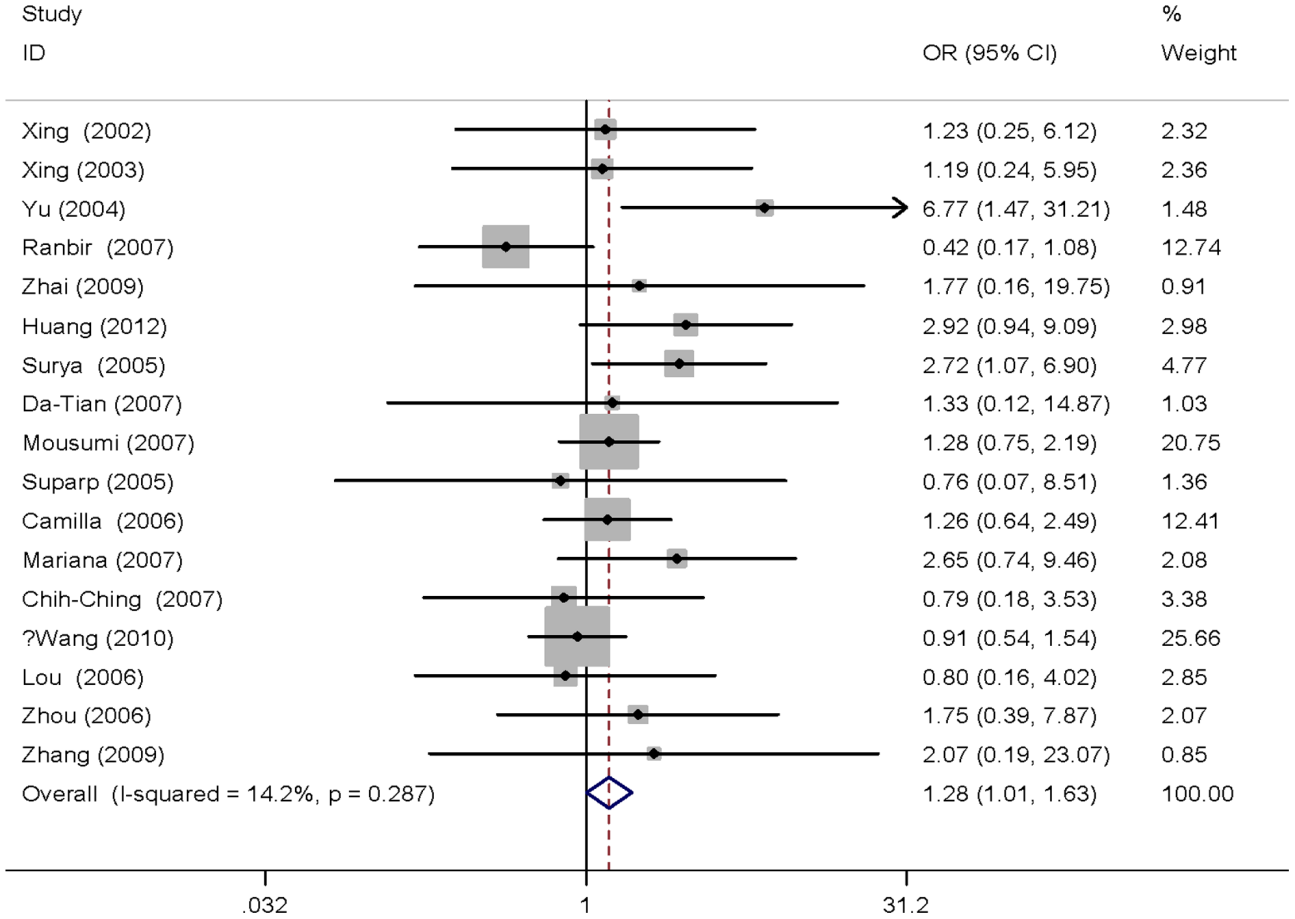
Forest plot of digestive cancer risk associated with the XPD Lys751Gln polymorphisms in Asian subgroups (based on homozygote comparison). A fixed-effects model was used. The squares and horizontal lines correspond to the study-specific OR and 95% CI. The area of the squares reflects the weight (inverse of the variance). The diamond represents the summary OR and 95% CI.

Table2 lists the main results of the meta-analysis for *XPD* Lys751Gln: having the Gln/Gln genotype is a risk factor for digestive tract cancers: GlnGln *vs*. LysLys: OR = 1.12, 95% CI = 1.01–1.24, *P* = 0.029, *P*_heterogeneity_ = 0.133. *I* ^2^ = 20.9% (Figure 2). We did not find any significant association between the other genetic models and digestive tract cancers. The results of stratified analysis by cancer type, source of controls and ethnicity were shown in table 2. The Gln/Gln vs. Lys/Lys genotype had an elevated risk in Asian population (OR = 1.28, 95% CI = 1.01–1.63, *P* = 0.045, *P*_heterogeneity_ = 0.287, *I* ^2^ = 14.2%; Figure 3). High heterogeneity was found in esophageal cancer and hospital-based studies, so the results can not be pooled together. In addition, the results did not suggest any association between *XPD* Lys751Gln polymorphism and digestive cancers susceptibility for all genetic models in European individuals or in population-based studies overall.

**Table 2:** Pooled ORs and 95%CIs of stratified meta-analysis.

Stratification	No.case/control	GlnGln *vs*.LysLys		GlnLys *vs*.LysLys		GlnGln+GlnLys *vs*.LysLys		GlnGln *vs*.GlnLys+LysLys	
		OR (95%CI)	*P*	OR (95%CI)	*P*	OR (95%CI)	*P*	OR (95%CI)	*P*
Total	40(9773/17185)	1.12(1.01,1.24)	0.029	1.04(0.98–1.11)	0.194	1.06(1.00,1.12)^b^	0.064	1.09(0.99,1.20)	0.072
Cancer type									
Colorectal cancer	11(3378/5319)	0.99(0.3,1.17)	0.870	0.99(0.89,1.09)	0.776	0.99(0.90,1.09)	0.790	1.00(0.85,1.17)	0.954
Gastric cancer	12(2542/5905)	1.05(0.85,1.29)	0.639	0.97(0.85,1.10)	0.612	0.98(0.86,1.11)	0.744	1.05(0.87,1.28)	0.630
Esophageal cancer	13(3096/5173)	1.29(1.08,1.54)^b^	0.005	0.90(0.81,1.00)^b^	0.056	0.91(0.77,1.07)^b^	0.235	0.84(0.66,1.07)	0.159
Oral cancer	4(757/688)	1.50(0.96,2.35)	0.078	0.88(0.60,1.30)	0.518	0.85(0.56,1.28)	0.430	0.72(0.47,1.12)	0.147
Ethnicity									
Asian	18(4669/6521)	1.28(1.01,1.63)	0.045	1.05(0.95,1.17)	0.340	1.08(0.98,1.19)	0.133	1.21(0.96,1.53)	0.110
European	22(5104/10564)	1.09(0.97,1.22)	0.144	1.04(0.96,1.12)	0.363	1.05(0.97,1.13)	0.232	1.07(0.96,1.19)	0.210
Source of control									
HB	20(5290/7075)	1.19(1.01,1.40)^b^	0.038	1.02(0.93,1.12)	0.703	1.02(0.89, 1.16)^b^	0.787	1.16(0.95,1.41)	0.140
PB	20(4483/10010)	1.08(0.94,1.23)	0.267	1.06(0.98,1.16)	0.157	1.07(0.98,1.15)	0.122	1.04(0.92,1.18)	0.715

NO: involved studies' number; *Gln Lys VS*.LysLys: Heterozygote comparison; GlnGln *vs*.LysLys: Homozygote comparison; GlnGln+GlnLys *vs*. LysLys: Dominant model; GlnGln *vs*. GlnLys+LysLys: Recessive model; Random model was chosen for data pooling when *P*<0.10 and/or *I^2^*>50%; otherwise fixed model was used.

b the results were excluded due to potential heterogeneity.

### Sensitivity analysis

**Figure 4:**
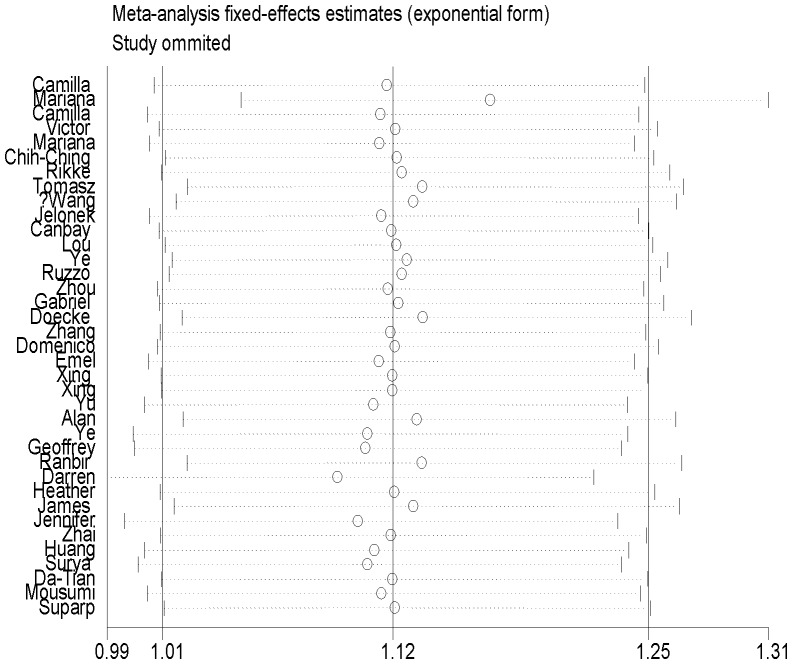
Influence analysis of the summary odds ratio coefficients on the association between XPD Lys751Gln homozygote comparison with digestive tract cancers risk. Results were computed by omitting each study (left column) in turn. Bars, 95% CI.

In the sensitivity analysis, when each particular study had been removed meta-analyses were conducted repeatedly. The corresponding pooled ORs were not qualitatively altered with or without this study. As shown in Figure 4, the most influencing single study on the overall pooled OR estimates seemed to be the one conducted by Mariana et al, which had a relatively large sample size. However, after the removal of the study, the result of the meta-analysis did not been influenced significantly: Gln/Gln vs. Lys/Lys: OR = 1.17, 95% CI: 1.05–1.30, indicating high stability of our results.

### Heterogeneity analysis

There was moderate heterogeneity among these studies in GlnGln+GlnLys *vs*.LysLys comparisons and Gln/Gln *vs*. Lys/Lys comparisons, but not in the other genetic models. We explored the source of heterogeneity for dominant model by cancer type, ethnicity, source of control, and found that esophageal cancer and hospital-based studies contributed to substantial heterogeneity (Table3). One reason may be that hospital-based studies had relatively small samples and were more prone to random error and false positive or negative results. Furthermore, it is very likely that the heterogeneity in esophageal studies and hospital-based studies are related since hospital-based studies predominate among the esophageal studies.

**Table 3:** Heterogeneity test.

Stratification	Gln Gln *vs*.LysLys	Gln Lys *vs*.LysLys	GlnGln+GlnLys *vs*.LysLys	GlnGln *vs*. GlnLys+LysLys
	Ph, I^2^ (%)	Ph, I^2^ (%)	Ph, I^2^ (%)	Ph, I^2^ (%)
Digestive cancers	0.133, 20.9	0.064,27.6	0.011, 38.3	0.385, 4.9
Cancer type				
Esophageal cancer	0.033, 46.6	0.022, 49.3	0.004,58.2	0.084,37.4
Gastric cancer	0.930,0	0.554,0	0.698,0	0.864,0
Colorectal cancer	0.310,13	0.470,0	0.328,12.2	0.387,5.9
Oral cancer	0.529, 0	0.095, 52.5	0.052,61.1	0.795, 0
Source of control				
Hospital-based	0.043,39.6	0.051,38.2	0.006,51.6	0.180,23.2
Population-based	0.550,0	0.243,17.3	0.184,22.3	0.715,0
Ethnicity				
Asian	0.287,14.2	0.174,24.2	0.057,38.0	0.353,8.6
European	0.137, 26.3	0.074,334	0.029,41.2	0.414,3.4

*Ph*: *P*-value of Q-test for heterogeneity identification; *I^2^* index: a quantitative measurement which indicates the proportion of total variation in study estimates that is due to between-study heterogeneity.

### Publication Bias

**Figure 5:**
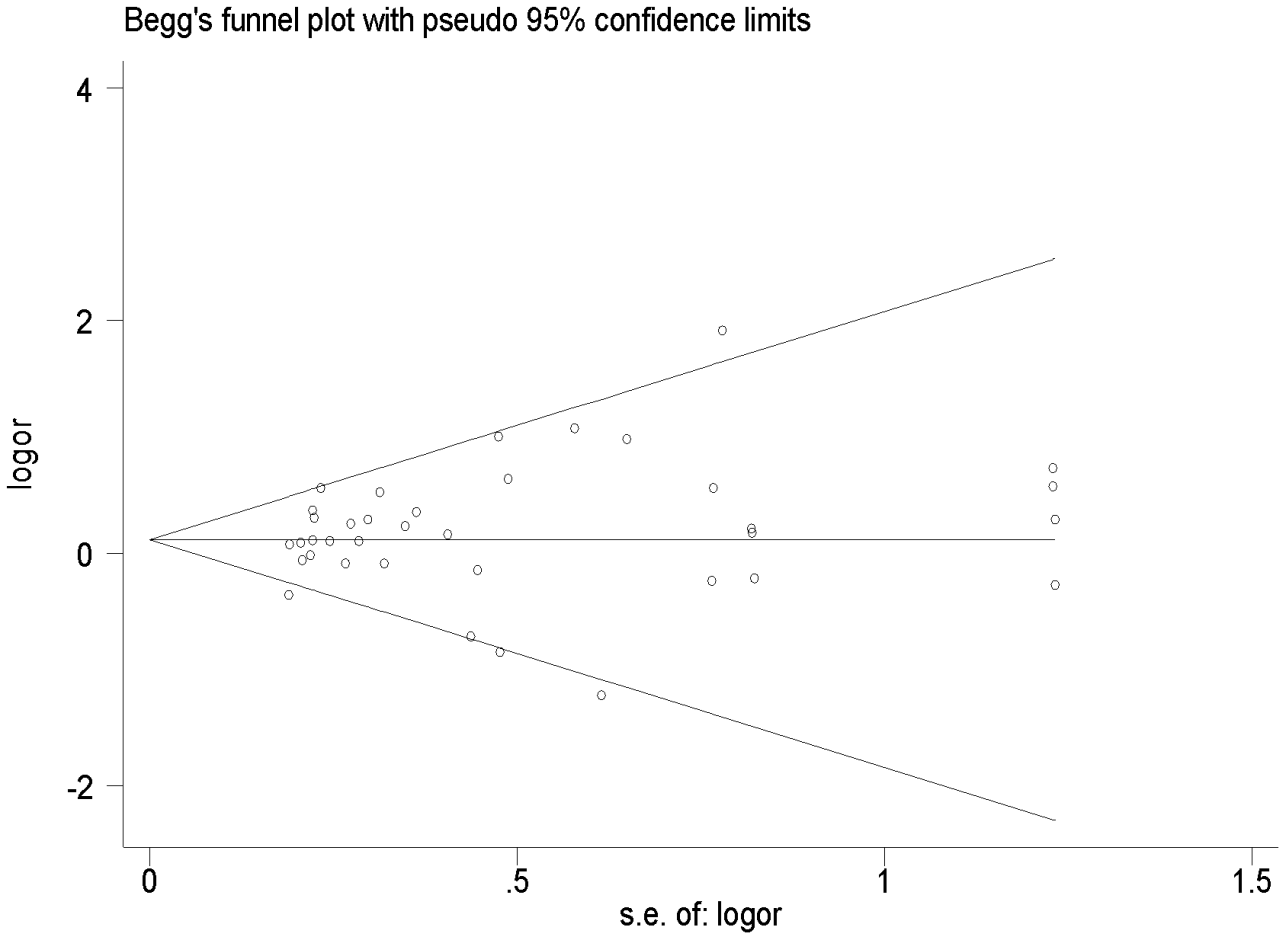
Begg's funnel plot for publication bias test (Homozygote comparison). Each point represents a separate study for the indicated association.

Begg's rank correlation method and Egger's weighted regression method were used to assess publication bias. There was no evidence of publication bias in *XPD* Lys751Gln (Begg's test *P* = 0.284, Egger's test *P* = 0.324, t = 1.00, 95% CI = 0.41–1.21). We present funnel plot for ORs of Gln/Gln versus Lys/Lys (Figure 5).

## Discussion

XPD plays a crucial role in NER, which is significant in the elimination of certain DNA cross-links, ultraviolet (UV) photo-lesions, and bulky chemical adducts. The *XPD* protein possesses both single-strand DNA-dependant ATP ase and 5′-3′ DNA helicase activities, which is essential for NER pathway and transcription [66]. Genetic variation in *XPD* may contribute to impaired DNA repair capacity and increased cancer risk. The Lys to Gln change at position 751 of *XPD* results in complete changes about the charge configuration of the amino acid, which affects the interactions of *XPD* protein and its helicase activator [67]. To date, a number of epidemiological studies have been conducted to evaluate the role of Lys751Gln polymorphism on several cancer risks, but the results remain controversial. As far as we know, several previous meta-analyses on *XPD* Lys751Gln polymorphism and cancers risk have been performed, such as gastric cancer, colorectal cancer, esophageal cancer, breast cancer and bladder cancer [14]–[23]. But to date, there is no meta-analysis on the association between digestive tract cancers risk and *XPD* Lys751Gln polymorphism. In order to derive a more precise estimation of relationship, we performed this meta-analysis of 37 studies, including 9027 cases and 16072 controls.

Through analyzing genotypes from the 37 eligible studies, we found the Gln/Gln genotype carries might be at potential risk to digestive tract cancers. The Lys to Gln variation on position 751 of *XPD* resulted in complete changes about the electronic configuration of the amino acid, which affected the interactions of *XPD* protein and its helicase activator [68]. Digestive tract cancers represent a homogenous group of malignancies in some ways. Different primary sites of digestive tract cancers have some shared risk factors. For example, except for smoking and alcohol consumption, eating rough, spicy, hot and non-digestible food is likely to damage the digestive tract tissue. In addition, *H.Pylori* infection is a major cause of gastric cancer, while nitrites derived from red meat and processed meat is a key risk factor for esophageal cancer and colorectal cancer. Such risk factors and their tissue specificity raise the possibility that the *XPD* polymorphism may be associated with digestive tract cancers risk. The functional *XPD* Lys751Gln polymorphism resulting in decreased activity of *XPD* protein may increase risk of digestive tract cancers on the basis of damage tissue.

In stratified analysis by cancer type, we found that all genetic models did not appear to have an effect on the risks of esophageal, gastric, colorectal and oral cancers. This was different from Ling Yuan's and Wu XB's studies [69], [70]. However Bo Chen et al. [71] detected that Gln/Gln genotype carriers might have an increased risk of gastric cancer in the Helico-bacter pylori (H.pylori)-positive population, but not in the Helico-bacter pylori (H. pylori)-negative population. One possible explanation is that the modulation of digestive tract cancers risk may depend not only on a single gene/single nucleotide polymorphism, but also on a joint effect of multiple polymorphisms within different genes or pathways, or on close interaction between polymorphisms and environmental factor. The other is that Helicobacter pylori infection is one of the clear etiologies of gastric cancer and maybe there is some relationship between helicobacter pylori and the polymorphic loci. In the subgroup of ethnicity, we found significant association between *XPD* Gln/Gln polymorphism and increased risks of digestive tract cancers in Asians but not in European. We think ethnic differences and diverse live environment may partly explain the phenomenon. Furthermore, we believed differences in diet, such as food structure and cooking way, were the main cause of this result. In addition, it was also likely that the observed ethnic differences may be due to chance because studies with small sample size may have insufficient statistical power to detect a slight effect or may have generated a fluctuated risk estimate [72].

In summary, this meta-analysis indicated that *XPD* Lys751Gln polymorphism, individuals carrying the variant homozygote Gln/Gln may increase the susceptibility of digestive tract cancers. And, significant associations were detected among Asians population. It should be noted explicitly: first, the effective sample size is much smaller for the Gln/Gln vs. Lys/Lys analyses than the other genetic models and therefore it is more prone to random error and false positive results; second, the results for GlnGln vs. GlyLys+LysLys, while not statistically significant (OR 1.09, 95% CI = 0.99–1.20, *P* = 0.072, *P*_heterogeneity_ = 0.385), strengthen our conclusion about which genetic model is most appropriate. Large-scale case-control and population-based association studies are warranted to validate the risk identified in the current meta-analysis and investigate the potential gene-gene and gene-environment interactions on digestive tract cancers risk.

## Supporting Information

Checklist S1 (DOC)
